# On Modern Progress in Ophthalmic Medicine

**Published:** 1894-12-15

**Authors:** Robert Brudenell Carter

**Affiliations:** Consulting Ophthalmic Surgeon to St. George's Hospital


### On Modern Progress in Ophthalmic Medicine and Surgery.

By Robert Brudenell Carter, F.R.C.S., Consulting Ophthalmic Surgeon to St. George's Hospital.

IRITIS—(*continued from p.* 111.)

Whatever may be the constitutional cause of iritis, the first manifestations of the disease are frequently, if not always, traceable to nervous irritation. I have seen a few cases at a period when no other symptoms were present. In these cases the pupil of the affected eye was contracted, and the patient complained of a feeling of local strain or dragging, such as sometimes follows the instillation of eserine. Generally speaking, however, this period has passed away before advice is sought, and the stage of effusion has commenced. When this is so, the conjunctiva will usually be slightly flushed, and the margin of the cornea will be surrounded by a narrow pinkish zone, due to distension of an annulus of fine blood-vessels situate in the sclera, just outside of the corneo-scleral junction. These vessels are too fine to be individually visible, but the colour effect which they produce is readily distinguishable from conjunctival redness by the application of gentle pressure with a finger tip placed outside the lower lid. Such pressure will for the moment empty the conjunctival vessels, but will have no effect upon the scleral plexus, the pink tinge of which will be rendered more conspicuous when the deeper red of the surface vascularity is diminished. At the same time, the surfaces of the iris and cornea will look somewhat dull by comparison with those of the unaffected eye, and the colour of the iris will be modified by the circumstance that the commencement of effusion has given a yellow tinge to the aqueous humour, through which a blue iris assumes a greenish aspect, while a brown one appears less bright than in health. The pupil will be either irresponsive to light, or sluggish in its movements, and the acuteness of vision will always be diminished. If atropine be instilled at this stage, its first effect will be to dilate the pupil irregularly, often into an outline bearing some resemblance to that of the club on a playing card, and thus to show that its margin has already contracted adhesions to the capsule of the lens. In such a condition, the instillation of atropine and cocaine will, in a large proportion of cases, cut short the disease without further treatment. The solution used for this purpose should contain a grain of neutral sulphate of atropine, and two grains of hydrochlorate of cocaine to two drachms of sterilised distilled water. A drop of this solution should be placed within the lower lid, repeated in five minutes, and again in another five minutes, and the threefold application should be made every four hours. In favourable cases, at the end of twenty-four hours the pupil will be fully dilated and circular, and the patient will be relieved of discomfort. All that is then required is to maintain dilatation by one application a day until the eye is once more of normal whiteness; and, in the meanwhile, to give both eyes functional rest, and to protect them from irritants and from exposure to cold.

When atropine and cocaine, used in the manner described, fail to render the pupil fully dilated and circular, or in any case in which the patient is not seen until the stage of effusion has existed for twenty-four hours or more, so that time has been given for the formation of adhesions of some tenacity, it will no longer be safe to trust to the mydriatic alone, and recourse should be had to mercury. In a case of great severity, notwithstanding the unpleasantness of the method, it is best to begin with inunction, and to rub in not less than a drachm of the strong blue ointment twice or even three times a day. In cases of more ordinary type, it will be sufficient to give blue pill, say two grains three times a day, and to combine with it a little opium. In either the mercury should be pushed until there is either distinct improvement in the eye or the commencement of a mercurial line upon the gums; and, if the latter should be the first to appear, the drug should be continued cautiously and in diminished doses in expectation of the former. When mercury is required, it will be necessary to insist upon extreme care in protecting the body from cold, and also, in view of the severity of the case, to carry out a regimen of greater strictness than is required in the milder examples of the disease. In both it is necessary to watch carefully over the period of convalescence, and especially over the return to visual work, an event which is not seldom attended by relapse, and which may call for a return to the methods of treatment originally required.

Iritis is sometimes complicated by an unusual amount and severity of pain, and this may be due to one of two causes, which must be carefully distinguished from each other. If it depend upon hyperæsthesia it must be controlled by morphia, which, for this purpose, is best given hypodermically. If it depend upon increased tension, due to excessive secretion within the eyeball, this will at once declare itself to the touch of educated fingers, and will, moreover, be attended with more impairment of sight than the appearance of the eye is calculated to explain. It calls imperatively for relief by paracentesis or by iridectomy. Of these proceedings, the former is generally to be preferred when the patient is near at hand, so that the little operation may be repeated if required; but recourse should be had to iridectomy whenever the assigned condition is not fulfilled. The severity of the attack in no way contra-indicates iridectomy, the operation being constantly followed by prompt and complete recovery.

When iritis is attended by an unusual degree of conjunctival vascularity, it is desirable to employ a leech or leeches, applied to the temple of the affected side, on the level of the eye, and as far away from it as the growth of hair will allow. A single leech, repeated daily or on alternate days, will generally be sufficient, and will afford great relief to the patient. It will also be well, as a rule, to commence the treatment by a purgative, and, whenever there is any indication of a rheumatic diathesis, I am accustomed to give salicylate of soda at bedtime, usually in a dose of twenty grains, taken in a tumblerful of hot water.

